# Determinants of health-related quality of life (HRQoL) among homeless individuals during the COVID-19 pandemic

**DOI:** 10.1007/s11136-023-03455-5

**Published:** 2023-07-11

**Authors:** Anna Brennecke, Fabian Heinrich, Victoria van Rüth, Katharina Dost, Wiebke Graf, Veronika Kowalski, Alessandra Rauch, Felicia Langenwalder, Klaus Püschel, Benjamin Ondruschka, Hans-Helmut König, Franziska Bertram, André Hajek

**Affiliations:** 1 Institute of Legal Medicine, University Medical Center Hamburg-Eppendorf, Hamburg, Germany; 2 Department of Health Economics and Health Services Research, University Medical Center Hamburg-Eppendorf, Hamburg, Germany

**Keywords:** Homeless, Health-related quality of life, EQ-5D, COVID-19

## Abstract

**Objective:**

Thus far, there is very limited knowledge regarding homeless individuals during the COVID-19 pandemic, particularly related to the health-related quality of life (HRQoL). Thus, our aim was to evaluate HRQoL and to clarify the determinants of HRQoL among homeless individuals during the COVID-19 pandemic in Germany.

**Methods:**

Data were taken from the national survey on psychiatric and somatic health of homeless individuals during the COVID-19 pandemic—NAPSHI (n = 616). The established EQ-5D-5L was used to quantify problems in five health dimensions, and its visual analogue scale (EQ-VAS) was used to record self-rated health status. Sociodemographic factors were included in regression analysis.

**Results:**

Pain/discomfort was the most frequently reported problem (45.3%), thereafter anxiety/depression (35.9%), mobility (25.4%), usual activities (18.5%) and self-care (11.4%). Average EQ-VAS score was 68.97 (SD: 23.83), and the mean EQ-5D-5L index was 0.85 (SD: 0.24). Regressions showed that higher age and having a health insurance were associated with several problem dimensions. Being married was associated with higher EQ-VAS scores.

**Conclusions:**

Overall, our study findings showed a quite high HRQoL among homeless individuals during the COVID-19 pandemic in Germany. Some important determinants of HRQoL were identified (e.g., age or marital status). Longitudinal studies are required to confirm our findings.

**Supplementary Information:**

The online version contains supplementary material available at 10.1007/s11136-023-03455-5.

## Introduction

Homelessness is an important challenge in Germany and beyond. Estimates from 2022 showed that about 263,000 people were homeless in Germany [1]. The number has increased in the last few years [2]. In the light of the COVID-19 pandemic, it is important to note that the population of homeless individuals is shown to have a higher risk for outbreaks of SARS-CoV-2 due to their living conditions such as crowded homeless shelters and limited access to hygienic supply [3]. In addition, measures to counteract the transmission of SARS-CoV-2 taken by the general population, as for example social distancing, contact tracing and personal hygienic precautions, are more difficult to apply for homeless individuals. These conditions make the population of homeless individuals even more vulnerable, which underlines the relevance to examine their situation during the COVID-19 -pandemic specifically.

Being homeless is associated with multimorbidity [4]. For example, disease prevalence in the categories infections, cardiovascular and respiratory conditions are higher among this population compared to the general adult population [4]. Moreover, homelessness is associated with a higher likelihood of mental health issues [4].

During the pandemic, studies indicate that homeless people are a particularly disadvantaged group when it comes to physical and mental health. For instance, a study reported high levels of depression amongst residents of homeless shelters compared to the general population during the COVID-19 pandemic in France [5]. Another study showed that homeless individuals have a higher mortality risk due to social inequality during the pandemic [6]. A study by Fields et al. found higher hospitalization rates due to COVID-19 itself for symptomatic homeless individuals compared to the general population [7].

Various studies have investigated the determinants of health-related quality of life (HRQoL) in general adult samples, whereas very little is known about the HRQoL in the population of homeless individuals, especially during the COVID-19-pandemic. A Swedish study evaluated the health status among homeless individuals in 2006 and 2018 and reported poor health in all EQ-5D-3L dimensions and the EQ VAS score in 2018 was even significantly lower than in 2006 [8]. In contrast to this, a study that investigated the HRQoL during the first wave of the pandemic (May/June 2020) in Hamburg (second largest city in Germany with now the highest number of homeless people nationally) among homeless individuals found remarkably high EQ-VAS scores particularly compared with the general population [9]. Also, higher age and lower education was positively associated with a lower EQ-5D-5L index.

For a better understanding it may be useful to describe the pandemic situation in Germany during the time of data collection. An incidence rate of about 14 cases per 100,000 population per week was measured in July 2021 (start of data collection). The incidence rate significantly increased in the following weeks, rising up to 74.7 cases per 100,000 population per week by the end of data collection in September 2021. During this period, Delta lineage of the virus became the predominant variant. At the time in Germany the 3G rule was applied (access to certain facilities and companies only for vaccinated, recovered or tested persons) in an effort to prevent further spreading of the coronavirus.

Due to the limited knowledge, the aim of this study was to describe HRQoL and to examine the determinants of HRQoL among the population of homeless individuals during later stages of the COVID-19 pandemic multicentrically. This is of particular relevance to address homeless individuals at risk of poor HRQoL during the COVID-19 pandemic. Moreover, this topic is important because HRQoL affects morbidity and mortality [10, 11].

## Methods

### Sample

Cross-sectional data were taken from the “national survey on psychiatric and somatic health of homeless individuals during the COVID-19 pandemic—NAPSHI”. The general aims of the NAPSHI were as follows: To assess the general life conditions of homeless individuals in Germany during the fourth wave.

**Fig. 1:**
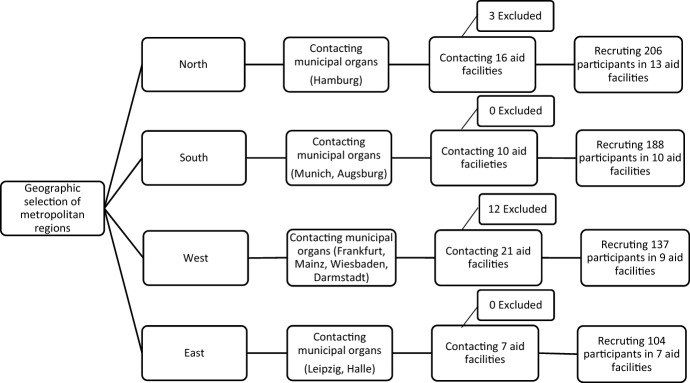
Flow chart

In this study, interviews were conducted by means of questionnaires in specialized medical practices, lodging houses, night shelters, women`s shelters or drug counselling centers. The data collection took place in the period from 26th July to 17th September 2021 mainly in the four large German cities Hamburg, Frankfurt am Main, Leipzig and Munich and in addition in the cities Mainz, Wiesbaden, Darmstadt, Halle and Augsburg. In sum, 39 different homeless aid institutions took part in the survey. Before the start of the study, all homeless aid institutions in the respective regions were contacted via the municipal administrative bodies. The project was presented to the municipal designated providers of the facilities. Principal inclusion criteria were a minimum age of 18 years, written informed consent and no permanent residence for more than 7 days and an informed consent to take part in the study. An existing pregnancy was the exclusion criteria. Another reason for exclusion from the study was when individuals did not want to be informed about results that could indicate potentially life-threatening diseases. In metropolitan regions, the number of individuals included were as follows: north (n = 206), south (n = 188), west (n = 137) and east (n = 104) of Germany. Please see the Flow Chart (Fig. 1) for further details.

In our analytical sample, n equaled 616 individuals. A compensation of five Euro per 30 min was offered to all participants. After questions about basic demographic information, we continued, among other things, with a questionnaire-based interview. The questionnaire was at the disposal in several languages (German, English, Russian, Polish and Bulgarian), translated by native speakers. In case the participants were able to read and understand the questions, they filled out the questionnaire themselves (i.e., written questionnaire). Otherwise, they were filled out via face-to-face interviews, in some cases with the help of a translator, which were either other participants or employees of the institutions. Written informed consent was provided by all participants prior to the investigation. The study was approved by the Ethics Committee of the Hamburg Chamber of Physicians (No.: PV7333).

### Dependent variables

In the present study, the EQ-5D-5L was used to evaluate the HRQoL. It is a well-validated instrument to quantify generic HRQoL (i.e., regardless of existing medical conditions). It considers five dimensions (mobility, self-care, usual activities, pain/discomfort and anxiety/depression) with five different levels in each case (no problems, slight problems, moderate problems, severe problems, extreme problems). Depending on the level, each dimension is assigned a digit, resulting in a combination of numbers consisting of 5 digits. For example, the health status `22333` indicates slight problems in mobility and self-care but moderate problems in usual activities, pain/discomfort and anxiety/depression. For each of the in total 5^5 = 3125 possible EQ-5D health states one index (EQ-5D index) can be calculated. This is done based on value sets derived from country-specific societal preferences. The German value set ranges from − 0.661 (extreme problems in all five dimensions) to 1 (no problems in any dimension). Since in the German value set negative values are possible, HRQoL conditions worse than death can be described. In this study, the responses were dichotomized due to skewness into the categories 0 (no problem) and 1 (slight problems, moderate problems, severe problems and extreme problems) for regression analysis.

Additionally, the questionnaire includes a visual analog scale (EQ VAS), which ranges from 0 to 100 points, with 0 points corresponding to the worst possible health status, while 100 points correspond to the best thinkable health status. It evaluates self-rated health according to the perception of the individuals.

### Independent variables

The following sociodemographic variables were included in regression analysis: age (in years), sex (male/female), marital status (married living together; other (including: married living permanently separated from spouse, single, widowed, divorced)) and level of education (distinguishing between: no qualification; grammar school/secondary modern school; vocational training qualification; university of applied sciences or university degree). In addition, the variable health-insurance coverage (not having health insurance; having health insurance) was considered.

### Statistical analysis

Initially, sample characteristics were described stratified by problems in the five dimension. After that, multiple logistic regressions were used to identify correlates of problems in EQ-5D dimensions. Afterwards, the determinants of EQ-VAS and EQ-5D index were analyzed using multiple linear regressions. A statistical significance level of p < 0.05 was set. The statistical analyses were conducted using Stata 16.0 (StataCorp., College Station, Texas, USA).

## Results

First, sample characteristics were depicted (stratified by problems in the five dimensions of the EQ-5D). Second, multiple logistic regressions and multiple linear regressions, as appropriate, were conducted to examine the determinants of the EQ-5D outcomes. Please see the next two sections.

### Sample characteristics

The total analytical sample included 616 participants with an average age of 43.4 (SD: 12.1), and about 18% of the individuals were female. About 4% were married and lived together. Secondary school education was reported by a total of 45% of the individuals, followed by 28% of the individuals having a tertiary school education. In sum, 67% of the individuals stated to have a health insurance. Average EQ-VAS score was 68.97 (SD: 23.83; skewness: − 0.65; kurtosis: 2.80), and the mean EQ-5D-5L index was 0.85 (SD: 0.24; skewness: − 2.09; kurtosis: 7.45). Please see the box-plots for further details (Supplementary Fig. 1 and Supplementary Fig. 2).

Table 1 illustrates the sample characteristics stratified by problems in the EQ-5D dimensions. Pain/discomfort was the most frequently reported problem (45.3%), thereafter anxiety/depression (35.9%), mobility (25.4%), usual activities (18.5%) and self-care (11.4%). For example, individuals having problems in the dimension mobility had a comparably high average age (47.4 years, SD: 13.1).

**Table 1:** Sample characteristics stratified by problems in EQ-5D dimensions

	Mobility		Self-care		Activities		Pain/discomfort		Anxiety/depression	
	No problems (n = 459)	Problems (n = 156)	No problems (n = 544)	Problems (n = 70)	No problems (n = 499)	Problems (n = 113)	No problems (n = 337)	Problems (n = 279)	No problems (n = 392)	Problems (n = 220)
Age	42.4 (11.5)	47.4 (13.1)	43.2 (11.9)	46.4 (12.8)	43.2 (11.8)	45.2 (13.0)	41.5 (11.6)	46.2 (12.3)	43.6 (11.8)	43.5 (12.7)
Sex										
Male	373 (74.3%)	129 (25.7%)	440 (88.0%)	60 (12.0%)	405 (81.0%)	95 (19.0%)	277 (55.2%)	225 (44.8%)	335 (67.1%)	164 (32.9%)
Female	81 (75.7%)	26 (24.3%)	99 (91.7%)	9 (8.3%)	88 (83.0%)	18 (17.0%)	56 (51.9%)	52 (48.1%)	54 (50.5%)	53 (49.5%)
Marital status^a^										
Married, living together	22 (84.6%)	4 (15.4%)	24 (92.3%)	2 (7.7%)	24 (92.3%)	2 (7.7%)	21 (80.8%)	5 (19.2%)	18 (72.0%)	7 (28.0%)
Other	423 (74.3%)	146 (25.7%)	503 (88.4%)	66 (11.6%)	457 (80.6%)	110 (19.4%)	306 (53.7%)	264 (46.3%)	365 (64.3%)	203 (35.7%)
Level of education										
No education	78 (72.9%)	29 (27.1%)	92 (84.4%)	17 (15.6%)	83 (77.6%)	24 (22.4%)	58 (53.7%)	50 (46.3%)	70 (64.8%)	38 (35.2%)
Primary	211 (78.1%)	59 (21.9%)	244 (90.7%)	25 (9.3%)	222 (82.8%)	46 (17.2%)	154 (56.8%)	117 (43.2%)	168 (62.7%)	100 (37.3%)
Secondary	127 (73.4%)	46 (26.6%)	154 (89.5%)	18 (10.5%)	141 (81.5%)	32 (18.5%)	90 (52.3%)	82 (47.7%)	115 (66.9%)	57 (33.1%)
Tertiary	28 (66.7%)	14 (33.3%)	35 (83.3%)	7 (16.7%)	34 (81.0%)	8 (19.0%)	21 (50.0%)	21 (50.0%)	23 (54.8%)	19 (45.2%)
Health insurance										
No	133 (70.7%)	55 (29.3%)	168 (88.4%)	22 (11.6%)	151 (80.7%)	36 (19.3%)	116 (61.4%)	73 (38.6%)	134 (71.3%)	54 (28.7%)
Yes	316 (76.5%)	97 (23.5%)	364 (88.8%)	46 (11.2%)	335 (81.5%)	76 (18.5%)	213 (51.6%)	200 (48.4%)	251 (61.2%)	159 (38.8%)

^a^Marital status: other (married living permanently separated from spouse, single, widowed, divorced)

### Regression analysis

In Table 2, findings of multiple logistic regressions are shown, with the outcome measures having problems in the dimensions mobility, self-care, activities, pain/discomfort and anxiety/depression.

**Table 2:** Determinants of problems in EQ-5D dimensions. Results of multiple logistic regressions

Independent variables	(1)	(2)	(3)	(4)	(5)
	Problems in mobility	Problems in dimension self-care	Problems in dimension usual activities	Problems in dimension pain/discomfort	Problems in anxiety/depression
Age	1.04*** (1.02–1.05)	1.02 + (1.00–1.04)	1.01 (1.00–1.03)	1.04*** (1.02–1.06)	1.00 (0.99–1.02)
Marital status^a^: other (reference: married and living together)	2.99 + (0.83–10.71)	1.58 (0.35–7.20)	2.75 (0.62–12.21)	4.33* (1.40–13.42)	1.16 (0.43–3.13)
Education^b^					
Secondary (ref: no education)	0.72 (0.42–1.23)	0.51 + (0.26–1.01)	0.71 (0.41–1.26)	0.79 (0.49–1.27)	1.07 (0.66–1.75)
Tertiary (ref: no education)	0.78 (0.44–1.39)	0.53 + (0.25–1.11)	0.69 (0.37–1.29)	0.74 (0.44–1.26)	0.92 (0.53–1.58)
University degree (ref: no education)	1.08 (0.48–2.44)	0.92 (0.34–2.50)	0.72 (0.29–1.82)	0.81 (0.38–1.76)	1.39 (0.64–2.99)
Health insurance: yes (ref: no)	0.64* (0.42–0.98)	0.91 (0.52–1.61)	0.91 (0.57–1.43)	1.32 (0.91–1.94)	1.53* (1.03–2.28)
Sex: Female (ref: Male)	0.99 (0.58–1.66)	0.60 (0.27–1.31)	0.92 (0.52–1.63)	1.11 (0.70–1.74)	1.88** (1.20–2.94)
Constant	0.07** (0.01–0.36)	0.11* (0.01–0.92)	0.08* (0.01–0.57)	0.03*** (0.01–0.12)	0.09** (0.02–0.38)
Observations	553	553	551	554	551
Pseudo R^2^	0.04	0.03	0.01	0.05	0.02

Odds Ratios are reported; 95% CI in parentheses; ***p < 0.001, **p < 0.01, *p < 0.05, + p < 0.10

^a^Marital status: other (married, living permanently separated from spouse; widowed; divorced)

^b^Education according to CASMIN classification

Regression analyses revealed that, among other things, higher age was associated with both a higher likelihood of mobility problems [OR 1.04, 95% CI 1.02–1.05] and problems in pain/discomfort [OR 1.04, 95% CI 1.02–1.06], whereas age was not associated with the other outcomes.

An existing health insurance was negatively associated with problems in mobility [OR 0.64, 95% CI 0.42–0.98], whereas it was positively associated with problems in anxiety/depression [OR 1.53, 95% CI 1.03–2.28]. Moreover, women had a higher likelihood of having problems in anxiety/depression compared to men [OR 1.88, 95%-CI 1.20–2.94].

In Table 3, findings of multiple linear regressions are shown with EQ-VAS and EQ-5D Index as outcome measures. Regressions revealed that a higher EQ-VAS is associated with being married and still being together (β = − 15.14, p < 0.001). Regression results (with EQ-5D Index as outcome measure) remained nearly the same in terms of significance (compared to the results with EQ-VAS as outcome measure). However, the association between secondary education (compared to no education) gained statistical significance (β = 0.07, p < 0.05).

**Table 3:** Determinants of EQ-VAS and EQ-5D-5L Index

Independent variables	EQ-VAS	EQ-5D-5L Index
Age	− 0.15 (0.09)	− 0.0019 + (0.0010)
Marital status^a^: other (reference: married and living together)	− 15.14*** (4.37)	− 0.1003* (0.0474)
Education^b^		
Secondary (ref: no education)	− 0.37 (2.74)	0.0674* (0.0325)
Tertiary (ref: no education)	2.35 (2.92)	0.0501 (0.0351)
University degree (ref: no education)	7.74 + (4.13)	0.0247 (0.0505)
Health insurance: yes (ref: no)	−1.33 (2.22)	− 0.0102 (0.0223)
Sex: Female (ref: Male)	− 2.15 (2.72)	− 0.0304 (0.0315)
Constant	93.09*** (7.29)	1.0286*** (0.0772)
Observations	558	543
R^2^	0.03	0.03

Results of multiple linear regressions

Unstandardized beta-coefficients are reported; robust standard errors in parentheses; ***p < 0.001, **p < 0.01, *p < 0.05, +p < 0.10

^a^Marital status: other (married, living permanently separated from spouse; widowed; divorced)

^b^Education according to CASMIN classification

In a robustness check, the main model was extended by adding the city as independent variable. The findings remained similar. These results can be found in Supplementary Table 1 and Supplementary Table 2.

Most of the independent variables had about 3–4% missing values and the dependent variables had about 6–7% missing values. Therefore, in a further robustness check, we used a full-information maximum likelihood approach [12] (instead of listwise deletion) in multiple linear regressions to deal with missing data. In sum, the findings remained similar. These findings are shown in detail in Supplementary Table 3.

## Discussion

The aim of this multicenter study was to describe HRQoL and determine factors associated with HRQoL of homeless individuals in a later stage of the COVID-19 pandemic. In sum, our study revealed a roughly comparable HRQoL among homeless individuals during the COVID-19 pandemic in Germany (average EQ-5D-5L index: 0.85, SD: 0.24)) compared to the general adult population nationally (average EQ-5D-5L index: 0.88, SD 0.18) in 2019 [13]. Also the average EQ-VAS score measured in our study (68.97, SD: 23.83), was similar to the average EQ-VAS score in the general adult population in Germany, which was 71.59 (SD: 21.36) in 2019 [13]. Considering the small amount of data of homeless individuals in general, our study, which is the largest homeless study in Germany, adds important knowledge to the factors being associated with HRQoL in this special cohort. Most of the preceding studies found the HRQoL among homeless people to be relatively low in comparison with the HRQoL of the general population [8, 14]. However, a cross-sectional study conducted in Hamburg by van Rüth et al. which investigated the HRQoL of homeless in May/June 2020 [9] also found a remarkably high HRQoL (average EQ-5D-5L index: 0.84, SD: 0.23)—which is well in line with our here presented findings.

The conditions of homeless individuals are certainly different from the general adult population. Thus, it is quite surprising that the average HRQoL is quite high. One possible explanation for the quite high HRQoL identified in our study could be a habituation of the homeless to their (health) conditions—which may be why their EQ-5D-5L index is similar to that of the general adult population although they are surrounded by special living conditions. Another possible reason could be that there might be a lower expectation within the group studied. Studies suggest that adverse childhood experiences is a risk factor for homelessness [15] and it may be the case that individuals who experienced adverse childhood experiences may have low expectations. The results of previous studies investigating the EQ-VAS among homeless somewhat vary, showing both low [8] and high [9] EQ-VAS scores. A Swedish study compared the EQ-5D-3L health status among homeless people in Stockholm in 2006 and 2018 [8], observing a mean EQ-VAS score of 53.4 among the homeless in 2018, which was not significantly different from the measured value a decade before. However, the EQ-VAS score determined by Van Rüth et al. [9] was 75.34 (SD: 22.23, ranging from 1 to 100). One reason for the inconclusive evidence could be that the study design (e.g., single center vs. multicenter studies; differences in sample size and period of data collection) varies between the studies.

Generally, as homeless individuals are often neglected in research, only scarce data exists concerning the HRQoL of individuals experiencing homelessness. Thus, our findings are rather difficult to compare with prior studies conducted among homeless individuals. However, the age association – which was found in our study—is in line with previous research [9]. It is also consistent with expectation as with age functional abilities decrease. This can result in an impairment of mobility and more pain/discomfort, as reported here.

The significantly higher likelihood of having problems in anxiety/depression in women compared to men in this study is consistent with the higher prevalence among women in the general adult population in Germany [16]. A study investigating depression during the COVID-19 pandemic amongst residents of homeless shelters in France also found that being female is associated with a higher likelihood of depression [5].

Being married and living together was associated with higher EQ-VAS as well as a higher EQ-5D-5L Index in our study. This is also well in line with prior research based on general population samples before the pandemic [17].

The negative correlation between existing health insurance and problems with mobility reported in our study could be the result of better access to medical treatment. Thus, individuals with health insurance may have more regular physician visits to treat their ailments. In contrast, having a health insurance was also significantly associated with a higher likelihood of having problems with anxiety/depression. This could be justified by the awareness of one`s physical state when in medical treatment, as in this cohort the prevalence of multimorbidity and chronic diseases is extremely high compared to the general population—as an Australian study investigating multimorbidity among people experiencing homelessness also found (75% and 68%) [18].

Some strengths and limitations are worth mentioning. There are only very few studies examining the health-related quality of life among homeless individuals, particularly during the COVID-19 pandemic. More precisely, this is the second study reporting on the HRQoL of homeless individuals during the COVID-19 pandemic using the EQ-5D questionnaire. Thus, this study markedly adds to the current knowledge concerning HRQoL among homeless individuals. One strength of the present study is its large sample. Moreover, this study is multi-centered, which adds to the generalizability. Another strength is the EQ-5D questionnaire, which is a common and valid tool to assess the HRQoL. Furthermore, our survey collected data regarding a special, vulnerable group, which is usually difficult to reach, because a considerable percentage of the suitable individuals might be aversed to participate due to illnesses, cognitive impairments, and general distrust of official institutions.

In contrast, some weaknesses should be noted. Although the overall sample was large, the proportion of women was relatively small, which should be acknowledged when interpreting the results for women separately. The proportion of homeless women in our sample may reflect the actual proportion of homeless women in Germany [1]. In addition, it may be difficult to generalize our findings. As our study only included homeless individuals who use certain social services, some selection bias might be present, as it may be difficult to transfer our findings to homeless individuals who do not make use of those institutional accommodations. Furthermore, participation bias and exclusion criteria might have led to the underrepresentation of individuals with bad physical and mental conditions. Also, the cross-sectional character makes it difficult to clarify the directionality between the factors examined in this study. As we contacted all homeless aid institutions in the respective regions and those who agreed to participate took part in the study, it was neither a random selection nor based on fixed criteria. Moreover, some aid facilities could not be included and a response rate could not be calculated. Thus, the generalizability of our results should be treated with caution. Furthermore, it should be noted that the questionnaires were translated by native speakers and therefore the gold standard of forward and backward translation was not carried out. Additionally, it may be of interest to examine the association between income (or amount of donations) and HRQoL among homeless individuals in future studies.

## Conclusion

Compared to the general adult population [19] during the COVID-19 pandemic, our study findings showed a quite high HRQoL among homeless individuals during the COVID-19 pandemic. Some determinants of HRQoL were identified (e.g., age or marital status).

This knowledge is important to characterize homeless individuals at risk for low HRQoL. In turn, this is important because HRQoL and subjective well-being affect morbidity and mortality [10, 11]. However, longitudinal studies are required to confirm our findings.

### Supplementary Information

Below is the link to the electronic supplementary material.

Supplementary file1 (PDF 52 KB)

Supplementary file2 (PDF 48 KB)

Supplementary file3 (DOCX 34 KB)

## Data Availability

The datasets analyzed during the current study are not publicly available due to ethical restrictions involving patient data but are available from the corresponding author on reasonable request.
