# Supplementary figures and images for: Determinants of health-related quality of life (HRQoL) among homeless individuals during the COVID-19 pandemic
Source: Qual Life Res. 2023 Jul 11;32(11):3075–83. doi: 10.1007/s11136-023-03455-5 (PMC10522712; doi:10.1007/s11136-023-03455-5)

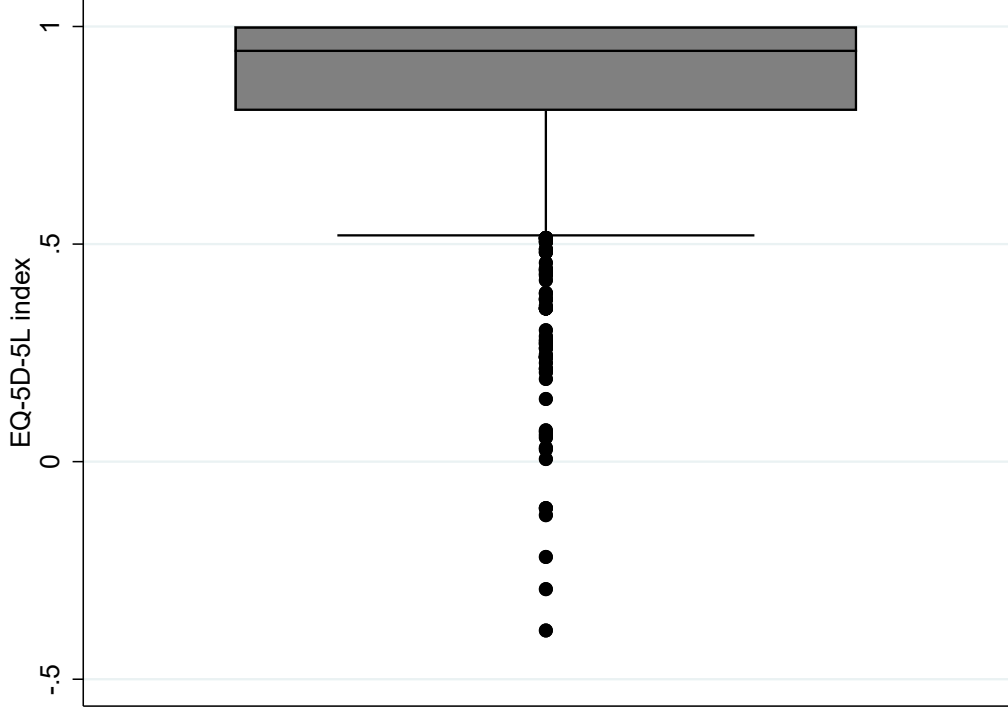

Supplement: Supplementary file 1 — Supplementary file1 (PDF 52 KB) [file 11136_2023_3455_MOESM1_ESM.pdf]

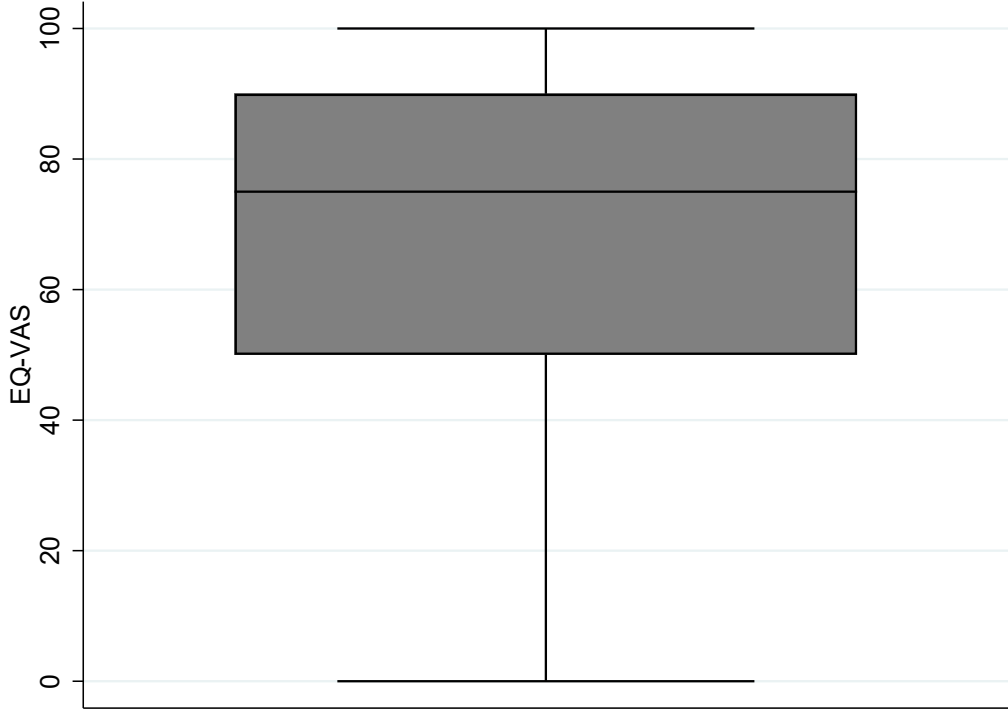

Supplement: Supplementary file 2 — Supplementary file2 (PDF 48 KB) [file 11136_2023_3455_MOESM2_ESM.pdf]
