# Supplementary material for: Determinants of health-related quality of life (HRQoL) among homeless individuals during the COVID-19 pandemic
Source: Qual Life Res. 2023 Jul 11;32(11):3075–83. doi: 10.1007/s11136-023-03455-5 (PMC10522712; doi:10.1007/s11136-023-03455-5)
Supplement: Supplementary file 3 — Supplementary file3 (DOCX 34 KB) [file 11136_2023_3455_MOESM3_ESM.docx]

Supplementary Table 1. Determinants of problems in EQ-5D dimensions. Results of multiple logistic regressions – additionally adjusting for city

|  | (1) | (2) | (3) | (4) | (5) |
| --- | --- | --- | --- | --- | --- |
| Independent variables | Problems in mobility | Problems in dimension self-care | Problems in dimension usual activities | Problems in dimension pain/discomfort | Problems in anxiety/depression |
| Age | 1.04*** | 1.03* | 1.01 | 1.04*** | 1.00 |
|  | (1.02 - 1.05) | (1.00 - 1.05) | (1.00 - 1.03) | (1.03 - 1.06) | (0.99 - 1.02) |
| Marital status^1^: other (reference: married and living together) | 3.04+ | 1.39 | 2.95 | 4.09* | 1.05 |
|  | (0.84 - 11.05) | (0.30 - 6.50) | (0.65 - 13.32) | (1.31 - 12.79) | (0.38 - 2.88) |
| Education^2^  - Secondary (ref: no education) | 0.72 | 0.49* | 0.71 | 0.78 | 1.09 |
|  | (0.42 - 1.24) | (0.25 - 0.99) | (0.40 - 1.25) | (0.48 - 1.27) | (0.66 - 1.78) |
| - Tertiary (ref: no education) | 0.83 | 0.51+ | 0.71 | 0.77 | 0.98 |
|  | (0.46 - 1.49) | (0.24 - 1.10) | (0.38 - 1.34) | (0.45 - 1.31) | (0.56 - 1.70) |
| - University degree (ref: no education) | 1.15 | 0.94 | 0.75 | 0.83 | 1.44 |
|  | (0.50 - 2.60) | (0.34 - 2.57) | (0.30 - 1.90) | (0.38 - 1.80) | (0.66 - 3.13) |
| Health insurance: yes (ref: no) | 0.63* | 0.81 | 0.88 | 1.27 | 1.53* |
|  | (0.41 - 0.96) | (0.45 - 1.47) | (0.55 - 1.42) | (0.86 - 1.88) | (1.02 - 2.31) |
| Sex: Female (ref: Male) | 1.01 | 0.60 | 0.94 | 1.13 | 1.96** |
|  | (0.59 - 1.70) | (0.27 - 1.33) | (0.53 - 1.66) | (0.72 - 1.79) | (1.25 - 3.08) |
| City: - Hamburg (ref: Frankfurt) | 1.11 | 1.46 | 0.87 | 1.31 | 1.56+ |
|  | (0.65 - 1.90) | (0.69 - 3.12) | (0.48 - 1.55) | (0.80 - 2.15) | (0.94 - 2.59) |
| - Leipzig | 1.08 | 2.21+ | 1.03 | 1.42 | 1.18 |
|  | (0.55 - 2.12) | (0.91 - 5.35) | (0.51 - 2.08) | (0.78 - 2.59) | (0.64 - 2.19) |
| - Munich | 0.61+ | 0.79 | 0.60 | 0.74 | 0.78 |
|  | (0.34 - 1.09) | (0.34 - 1.83) | (0.32 - 1.12) | (0.44 - 1.22) | (0.46 - 1.33) |
| Constant | 0.07** | 0.10* | 0.10* | 0.02*** | 0.08*** |
|  | (0.01 - 0.37) | (0.01 - 0.90) | (0.01 - 0.66) | (0.00 - 0.11) | (0.02 - 0.34) |
| Observations | 553 | 553 | 551 | 554 | 551 |
| Pseudo R² | 0.05 | 0.04 | 0.02 | 0.06 | 0.03 |

Odds Ratios are reported; 95% CI in parentheses; *** p<0.001, ** p<0.01, * p<0.05, + p<0.10; ^1^Marital status: other (married, living permanently separated from spouse; widowed; divorced); ^2^Education according to CASMIN classification; Participants in Mainz and Wiesbaden were assigned to the city of Frankfurt and participants in Augsburg were assigned to Munich

Supplementary Table 2. Determinants of EQ-VAS and EQ-5D-5L Index. Results of multiple linear regressions – additionally adjusting for city

| Independent variables | EQ-VAS | EQ-5D-5L Index |
| --- | --- | --- |
| Age | -0.17+ | -0.0022* |
|  | (0.09) | (0.0010) |
| Marital status^1^: other (reference: married and living together) | -13.39** | -0.0830+ |
|  | (4.25) | (0.0462) |
| Education^2^  - Secondary (ref: no education) | -0.74 | 0.0670* |
|  | (2.73) | (0.0318) |
| - Tertiary (ref: no education) | 2.31 | 0.0462 |
|  | (2.98) | (0.0349) |
| - University degree (ref: no education) | 7.94+ | 0.0242 |
|  | (4.33) | (0.0521) |
| Health insurance: yes (ref: no) | -1.96 | -0.0070 |
|  | (2.26) | (0.0236) |
| Sex: Female (ref: Male) | -1.94 | -0.0326 |
|  | (2.73) | (0.0314) |
| City: - Hamburg (ref: Frankfurt) | -6.94* | -0.0749** |
|  | (2.82) | (0.0289) |
| - Leipzig | 0.70 | -0.0442 |
|  | (3.08) | (0.0342) |
| - Munich | -5.37+ | -0.0033 |
|  | (2.97) | (0.0264) |
| Constant | 93.09*** | 1.0555*** |
|  | (7.29) | (0.0751) |
|  |  |  |
| Observations | 557 | 543 |
| R² | 0.05 | 0.05 |

Unstandardized beta-coefficients are reported; robust standard errors in parentheses; *** p<0.001, ** p<0.01, * p<0.05, + p<0.10; ^1^Marital status: other (married, living permanently separated from spouse; widowed; divorced); ^2^Education according to CASMIN classification; Participants in Mainz and Wiesbaden were assigned to the city of Frankfurt and participants in Augsburg were assigned to Munich

Supplementary Table 3. Determinants of EQ-VAS and EQ-5D-5L Index. Results of multiple linear regressions (with full-information maximum likelihood to address missing values)

| Independent variables | EQ-VAS | EQ-5D-5L Index |
| --- | --- | --- |
| Age | -0.15+ | -0.0019* |
|  | (0.09) | (0.0009) |
| Marital status^1^: other (reference: married and living together) | -13.22** | -0.0782 |
|  | (4.07) | (0.0495) |
| Education^2^  - Secondary (ref: no education) | -0.38 | 0.0614+ |
|  | (2.66) | (0.0314) |
| - Tertiary (ref: no education) | 2.98 | 0.0508 |
|  | (2.85) | (0.0336) |
| - University degree (ref: no education) | 8.04* | 0.0294 |
|  | (4.04) | (0.0479) |
| Health insurance: yes (ref: no) | -1.26 | -0.0136 |
|  | (2.12) | (0.0216) |
| Sex: Female (ref: Male) | -1.85 | -0.0339 |
|  | (2.54) | (0.0297) |
|  |  |  |
| Constant | 91.19*** | 1.0199*** |
|  | (6.82) | (0.0757) |
|  |  |  |
| Observations | 659 | 660 |
| R² | 0.03 | 0.02 |

Unstandardized beta-coefficients are reported; robust standard errors in parentheses; *** p<0.001, ** p<0.01, * p<0.05, + p<0.10; ^1^Marital status: other (married, living permanently separated from spouse; widowed; divorced); ^2^Education according to CASMIN classification
